# Drought Disrupts Auxin Localization in Abscission Zone and Modifies Cell Wall Structure Leading to Flower Separation in Yellow Lupine

**DOI:** 10.3390/ijms21186848

**Published:** 2020-09-18

**Authors:** Aleksandra Bogumiła Florkiewicz, Agata Kućko, Małgorzata Kapusta, Sebastian Burchardt, Tomasz Przywieczerski, Grażyna Czeszewska-Rosiak, Emilia Wilmowicz

**Affiliations:** 1 Department of Plant Physiology and Biotechnology, Nicolaus Copernicus University, 1 Lwowska Street, 87-100 Toruń, Poland; 279382@stud.umk.pl (A.B.F.); 282371@stud.umk.pl (S.B.); 289337@stud.umk.pl (T.P.); wiktoria@umk.pl (G.C.-R.); 2 Department of Plant Physiology, Institute of Biology, Warsaw University of Life Sciences-SGGW (WULS-SGGW), Nowoursynowska 159 Street, 02-776 Warsaw, Poland; kuckoa@poczta.onet.pl; 3 Department of Plant Cytology and Embryology, University of Gdańsk, 59 Wita Stwosza, 80-308 Gdańsk, Poland; malgorzata.kapusta@biol.ug.edu.pl

**Keywords:** abscission zone, auxin, cell wall, drought, pectin, phytohormones, yellow lupine, yielding

## Abstract

Drought causes the excessive abscission of flowers in yellow lupine, leading to yield loss and serious economic consequences in agriculture. The structure that determines the time of flower shedding is the abscission zone (AZ). Its functioning depends on the undisturbed auxin movement from the flower to the stem. However, little is known about the mechanism guiding cell–cell adhesion directly in an AZ under water deficit. Therefore, here, we seek a fuller understanding of drought-dependent reactions and check the hypothesis that water limitation in soil disturbs the natural auxin balance within the AZ and, in this way, modifies the cell wall structure, leading to flower separation. Our strategy combined microscopic, biochemical, and chromatography approaches. We show that drought affects indole-3-acetic acid (IAA) distribution and evokes cellular changes, indicating AZ activation and flower abortion. Drought action was manifested by the accumulation of proline in the AZ. Moreover, cell wall-related modifications in response to drought are associated with reorganization of methylated homogalacturonans (HG) in the AZ, and upregulation of pectin methylesterase (PME) and polygalacturonase (PG)—enzymes responsible for pectin remodeling. Another symptom of stress action is the accumulation of hemicelluloses. Our data provide new insights into cell wall remodeling events during drought-induced flower abscission, which is relevant to control plant production.

## 1. Introduction

One of the many adverse effects of climate change induced by global warming is soil water deficit [1,2]. Drought stress, as the main abiotic factor, not only negatively regulates plant growth and development but also significantly affects crop yield, which have important agronomic consequences. Legumes (*Fabaceae*), including yellow lupine (*Lupinus luteus* L.), are characterized by a strong correlation between weather conditions and yield quality and/or quantity [3]. As we have recently shown in this species, drought activates a specialized, spatially organized structure responsible for organ detachment, which is called the abscission zone (AZ) [4,5]. This causes premature abortion of flowers, which generates huge economic losses. We have also pointed out that water deficit upregulates the expression of the elements involved in the molecular abscission-associated pathway (*LlIDL – INFLORESCENCE DEFICIENT IN ABSCISSION, LlHAE – HAESA, LlMPK6 – MITOGEN-ACTIVATED PROTEIN KINASE6*) locally in AZ cells [3,5]. Moreover, water loss disrupts the redox balance, which is reflected by an increasing hydrogen peroxide (H_2_O_2_) level and catalase (CAT) activity, directly in the place of flower detachment [3,6]. At the same time, biosynthesis of the main hormonal stimulators of flower abscission, abscisic acid (ABA), and ethylene (ET) is stimulated [3,5]. As we have already proven, the functioning of the lupine AZ and, as a consequence, flower separation is also determined by a spatial and temporal distribution of indole-3-acetic acid (IAA) in different regions of the AZ, which is maintained by the interactions with ET [6,7]. In turn, the ET role, as the main effector of abscission, is to activate hydrolytic enzymes, responsible for cell wall remodeling that causes middle lamella hydrolysis and, finally, organ separation [8,9,10]. 

Degradative processes accompanying organ abscission are also connected with the redistribution of cell wall components [11,12,13]. Cell adhesion in the AZ is highly dependent on the modification of the cell wall, which is 90% built of polysaccharides. Among them, the majority of plant cell walls are cellulose chains organized in microfibrils and branched hemicelluloses, as well as pectin [14]. Among all pectin, the most important are homogalacturonans (HG), rhamnogalacturonans I (RG-I), and rhamnogalacturonans II (RG-II) [15,16]. HGs form a middle lamella and are a matrix with suspended cellulose elements. Importantly, the stability of middle lamella and mechanical properties of the cell wall are modulated by the degree and pattern of HG methyl-esterification [15,16]. HGs synthesized de novo are rich in methyl residues connected to the polygalacturonic acids, which ensures their greater liquidity. Demethylation of HG allows for the formation of cross-links with calcium ions (Ca^2+^) and the obtaining of an egg-box-like configuration between adjacent polymers [17]. Pectin demethylation is catalyzed by pectin methylesterase (PME, EC 3.1.1.11), which can participate both in the stiffening and loosening of middle lamella [11]. PME provides a substrate for polygalacturonase (PG), characterized by a high affinity for de-esterified galacturonic acid residues. PG hydrolyzes glycosidic bonds, resulting in the release of its monomers, dimers, or oligomers, leading to middle lamella disintegration [18,19,20,21,22].

Roongsattham et al. [23] suggested the dynamics of AZ-specific cell wall modifications for particular changes in the pectin portion/section and downregulation of methyl-esterified HG following abscission of oil palm fruit (*Elaeis guineensis* Jacq.). However, current knowledge about the molecular mechanisms evoked by water deficit in the organ AZ of crop species is rather scarce. Thus, our current challenge is to identify key regulatory molecules responsible for drought-induced cell wall modifications in yellow lupine. Overall, analyzing our previous results, available literature data, and bearing in mind the importance of water deficit as the main limiting factor for many crops’ productivity, here, we decided to check the hypothesis that drought disturbs the IAA balance around the AZ, leads to the specific structural changes of AZ cells, changes the pectin methylation degree that causes cell wall remodeling, disrupts cell-to-cell adhesion, and finally causes organ abscission.

## 2. Results

### 2.1. Drought Activates AZ Cells and Causes Proline Accumulation

In the beginning, we aimed to verify if the drought stress conditions are sufficient to induce proper response and activation of AZ cells in yellow lupine flowers. As shown in Supplementary Figure S1C,E,F, soil drought leads to histological changes indicating AZ cells activation, such as protoplast plasmolysis (Supplementary Figure S1D–F), and numerous cytoplasmic grains and aggregates (Supplementary Figure S1E,F) also located at the site of newly formed cell walls (Supplementary Figure S1D–F). The most spectacular symptoms confirming AZ activation are disorders in adhesion between adjacent cells and disruption of the tissue connection (Supplementary Figure S1D). Such changes were not observed in the inactive AZ (Supplementary Figure S1A,B).

**Figure 1:**
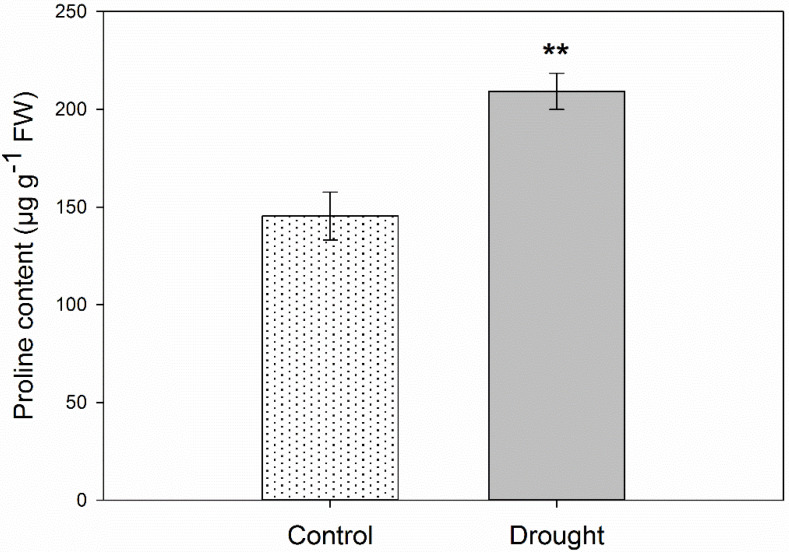
Soil drought stress increases proline content in flower abscission zone (AZ) of yellow lupine. Lupines were cultivated under water deficit conditions (25% WHC), while control plants were grown in the soil of optimal moisture (70% WHC). For analysis, sections of the abscission zone (AZ) were collected on the 48th day of cultivation. Data are presented as averages ± SE (*n* = 3). Significant differences in stressed plants in comparison to control are ** *p* < 0.01 (Student’s *t*-test).

In the next step, we analyzed the level of proline as a significant molecule playing a highly beneficial role in plants exposed to various adverse environmental stress conditions [24]. The drought was a stimulating factor for proline accumulation in the AZ. Its level was almost 40% higher in comparison to the inactive AZ (Figure 1). 

### 2.2. Soil Water Deficit Affects IAA Localization in Floral AZ

**Figure 2:**
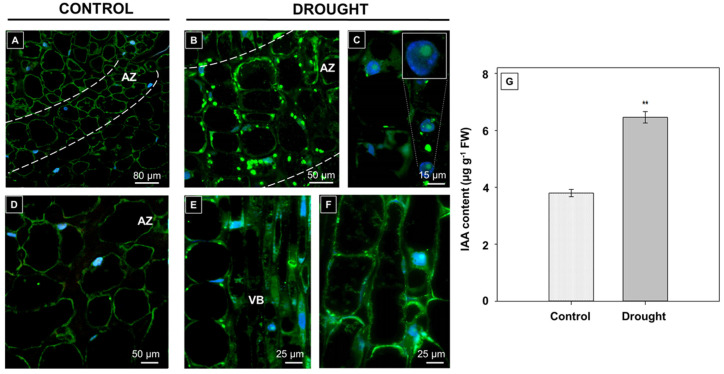
Drought stress affects indole-3-acetic acid (IAA) localization in abscission zone (AZ) cells**.** Immunolocalization of IAA in the floral AZ of yellow lupine exposed to drought-stress (25% WHC, water deficit conditions) (**B**,**E**,**F**) and AZ from control plants growing in the soil of optimal moisture (70% WHC) (**A**,**B**). Green fluorescence indicates IAA presence, whereas blue labeling corresponds to nuclei stained with DAPI. AZ area is marked by a white dotted line (**A**,**B**). Image (**D**) presents the magnified control AZ area presented in (**A**). IAA is found in nuclei (**C** insert) and cells located near the vascular bundles (VB) (**E**,**F**)**.** Bars are given in each image. Endogenous content of IAA in control and drought-treated AZs (**G**). For all analysis, sections of AZs were collected on the 48th day of cultivation. Data are presented as averages ± SE (*n* = 3). Significant differences in the stressed plants in comparison to control plants are ^**^ *p* < 0.01 (Student’s *t*-test).

One of the most important phytohormones involved in abscission-related processes in yellow lupine flowers is auxin [6,7]. At the same time, auxins are crucial players in cell wall remodeling events as they promote divisions, growth, and differentiation [25]. Thus, in this paper, we analyzed the impact of drought on the presence of IAA directly in AZ cells. In stressed plants, the fluorescence indicating IAA presence in AZ cells seems to be greater than in the control (Figure 1). The hormone was observed in the cytosol of AZ cells (Figure 2B). Specifically, IAA was localized mainly in the round compartments dotted in the peripheral cellular areas (Figure 2B). Interestingly, IAA was also detected in some nucleus (Figure 2C). Furthermore, diffuse fluorescence was found throughout the cells located near the vascular bundles (Figure 2E,F); however, in this case, the signal was noticed in the whole cytoplasm (Figure 2F). In the control section, IAA presents only in the boundary, thin layer of cytoplasm (Figure 2A,B). The chromatography analysis confirmed that drought increased the IAA level in the AZ cells (Figure 2G).

### 2.3. Drought Induces Changes in the Composition of Cell Wall Components

**Figure 3:**
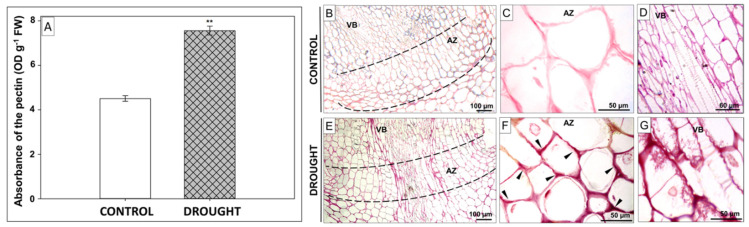
Drought causes changes in pectin distribution in floral abscission zone (AZ) of yellow lupine. Lupines were cultivated under water deficit conditions (25% WHC), while control plants were grown in the soil of optimal moisture (70% WHC). Sections of floral AZs were collected on the 48th day of cultivation. The total content of pectin based on pectin absorbance (**A**). Data are presented as averages ± SE (*n* = 3). Significant differences in the stressed plant in comparison to control plants are ** *p* < 0.01 (Student’s *t*-test). De-esterified pectin staining using Ruthenium red in the control AZ (**B**–**D**) and drought-stressed (**E**–**G**). AZ area is marked by the black dotted line (**B**,**E**). Arrowheads indicate the place of de-esterified pectin accumulation. Abbreviation: VB—vascular bundles. Bars are given in each image.

Organ abscission required significant modifications of cell wall components [10]. The next analysis revealed that AZ cells accumulated pectin in response to soil water deficit (Figure 3A). Important for cell adhesion is not only the total content of pectin synthesized in the Golgi apparatus but also the degree of their esterification [23]. Thus, in the next step, we used Ruthenium red to distinguish HG composition. It seems that the cell walls in the area of the AZ subjected to drought stress showed a higher red color intensity of the middle lamellas under the influence of Ruthenium red (Figure 3E–G) in relation to plants grown in optimal humidity conditions (Figure 3B–D). As the observed changes may be a manifestation of the accumulation of de-esterified pectin, in the next stage, the pattern of methylated pectin localization was analyzed in detail.

**Figure 4:**
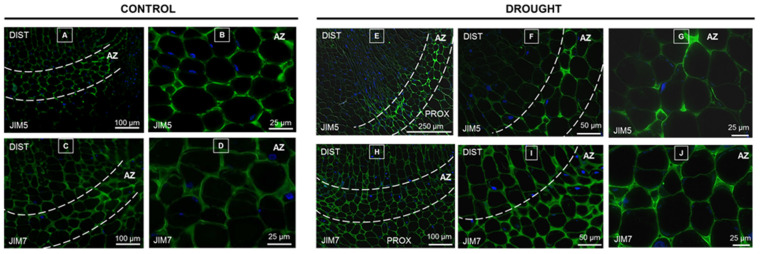
Drought leads to redistribution of low- and high-methylated pectin in the abscission zone (AZ) area. Immunolocalization of pectin in the flower AZ of yellow lupine cultivated under soil drought conditions (25% WHC, water deficit conditions) (**E**–**J**) and AZ of control lupines growing in the soil of optimal moisture (70% WHC) (**A**–**D**). For analysis, sections of AZs (marked by the white dotted line) were collected on the 48th day of cultivation. Low-methylated and un-methylated homogalacturonans (HGs) were detected by JIM5-Ab, while JIM7-Ab was used to localize high-methylated HGs. Green fluorescence indicates the pectin presence, while the blue signal corresponds to nuclei stained with DAPI. Images (**B**,**D**) are magnifications of AZ areas presented in (**A**,**C**)**,** respectively. Images (**F**,**G**) are magnified regions presented in (**E**). Images (**I**,**J**) are magnified regions presented in (**H**). Abbreviations: DIST—distal region, flower pedicel fragment above the AZ; PROX—proximal region, stem fragment below the AZ. Bars are given on each image.

We used specific antibodies to detect low- (31–40%) and non-methylated HGs (JIM5), as well as high-methylated (15–80%) HGs (JIM7) [26]. The immunocytochemical analysis showed a differential distribution of both types of pectin in the flower AZ of plants subjected to drought stress (Figure 4E–G) and in control AZ sections (Figure 4A,B). Importantly, drought conditions caused an accumulation of low-methylated HGs only specifically in the AZ cells (Figure 4E,F). In this case, the JIM5 signal was almost non-detectable in distal and proximal parts of the AZ area (Figure 4E–G). On the other hand, when plants were cultivated under optimal soil water content, the JIM5 signal was visible in the cell walls within the AZ area (Figure 4A,B), as well as above and below this structure (Figure 4A). High-methylated HGs were accumulated stronger in the drought-induced AZ (Figure 4H–J) compared to the control one (Figure 4C,D). The JIM7 signal was noted also in the distal and proximal AZ areas of both drought-treated (Figure 4H–J) and control plants (Figure 4C,D).

**Figure 5:**
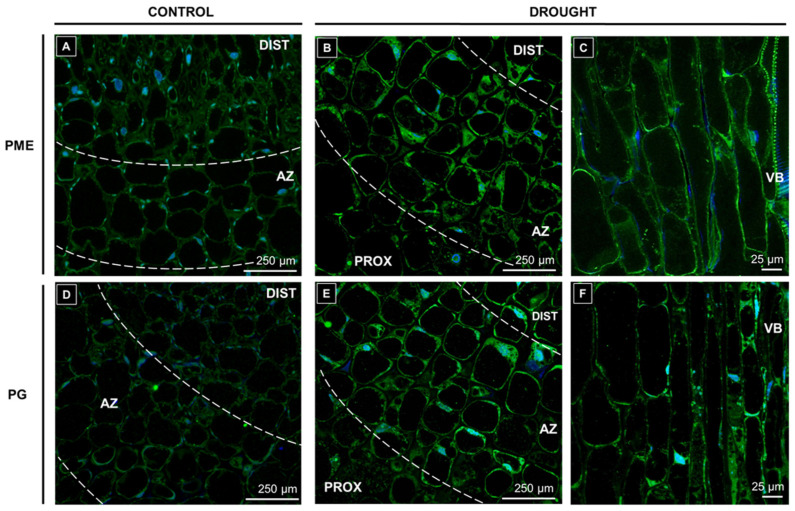
Drought influences the presence of pectin remodeling enzymes in the abscission zone (AZ) area. The impact of soil drought stress (25% WHC, water deficit conditions) on the localization of pectin-methylesterase (PME) (**B**,**C**) and polygalacturonase (PG) (**E**,**F**) was analyzed in the flower AZ of yellow lupine. Control was AZs excised from plants growing in the soil of optimal humidity (70% WHC) (**A**,**D**). For analysis, sections of AZs were collected on the 48th day of cultivation. Fixed material was dissected and incubated with monoclonal anti-PME-Ab (**A**–**C**) and anti-PG-Ab (**D**–**F**). Green fluorescence indicates the presence of the enzymes. Nuclei were stained with DAPI (blue fluorescence). AZ area is marked by white dotted lines (**A**,**B**,**D**,**E**). Abbreviations: DIST—a distal region of AZ, flower pedicel fragment above the AZ, PROX—a proximal region of AZ, stem fragment below the AZ, VB—vascular bundles. Bars are given in images.

Cell wall-remodeling enzymes are key players in abscission processes; thus, in this work, special attention has been given to check the influence of drought on the presence of PG and PME in the AZ area. For this purpose, we made an immunofluorescence experiment, which has shown the AZ-specific accumulation of both PG and PME in response to drought (Figure 5). The presence of PME was observed in the peripheral areas of the cytoplasm of AZ cells (Figure 5B). A weaker signal was noted in the cells neighboring the vascular bundle (Figure 5C); however, it was stronger than in the control section (Figure 5A). In the drought-treated AZ, a fluorescence signal indicating PG accumulation was observed in the cytoplasm (Figure 5E). Moreover, this enzyme was detected in vascular bundles (Figure 5F). Control floral AZ sections for both PME (Figure 5A) and PG (Figure 5B) showed an almost undetectable green signal. 

**Figure 6:**
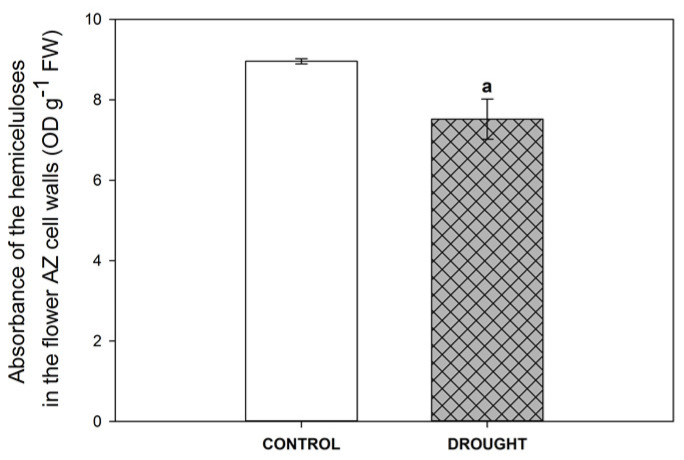
Hemicellulose content is negatively regulated by soil water deficit. The effect of drought stress (25% WHC, water deficit conditions) on the absorbance of hemicelluloses in the flower abscission zone (AZ) of yellow lupine was analyzed. Control AZ fragments were collected from plants growing in the soil of optimal conditions (70% WHC). For analysis, sections of AZs were harvested on the 48th day of cultivation. Data are presented as averages ± SE (*n* = 3). Significant differences in the stressed plants in comparison to control plants are ^a^ *p* < 0.05 (Student’s *t*-test).

Given that the hemicelluloses are dominant carbohydrates in the compound middle lamella, it was essential to investigate the influence of drought on the level of these molecules in the floral AZ. The results of the presented study clearly indicate that soil water deficit decreased the level of hemicellulose in AZ cells (Figure 6).

## 3. Discussion

The perception of drought stimuli induces a signal cascade governed by phytohormones, which are key elements that inform the whole plant about stress conditions. At the same time, hormones coordinate specific changes occurring in the AZ cells, which determine the time of organ separation. Water deficit in the soil is the factor causing primary osmotic disorders in plant cells, which generates changes leading to turgor loosening [27]. Then, a defensive response related to the production of osmoprotectants, including proline, is induced [28]. Observed here, increased content of proline in the lupine AZ (Figure 2D) indicates that a stress stimulus was received and the defense mechanism was switched on, which was also supported by cellular changes observed in the AZ area (Supplementary Figure S1C,E,D,F). These modifications are similar to those previously described as specific for drought-treated AZ cells [3]. Proline is a reservoir of carbon and nitrogen that ensures that the cell metabolic activity is maintained under adverse conditions [29,30,31]. Water deficit causes a slight accumulation of this amino acid in the beginning, followed by a drastic increase and, later, significant downregulation of its concentration [32]. In turn, osmotic stress, triggered by salinity in *Populus tremula*, quickly induces proline synthesis [33], similarly as in the case of *A. thaliana* [34] and *Vicia faba* [35]. As shown in apple leaves, a factor responsible for proline accumulation under drought is ABA, which activates antioxidant response [36]. Therefore, observed in lupine proline, upregulation in AZ cells (Figure 2D) could be evoked by drought-dependent ABA accumulation, which was proven in our previous work [3]. Proline, as a high hydrophilic amino acid, stabilizes the cell membrane structure and protects structural proteins and enzymes against denaturation, controls the pH of the cytosol, plays an antioxidant role, and neutralizes ROS (reactive oxygen species) [37], which are formed and detoxified in AZ cells in response to water deficit, as was already presented in lupine [3].

In our recent report, we determined the precise localization of IAA in the floral AZ of yellow lupine [6]. Further investigation revealed the presence of asymmetrical changes in both parts of the AZ structure in response to the disruption of polar auxin transport across the AZ, which is a factor evoking flower separation [7]. It is generally accepted that the higher level of IAA above the AZ (in the pedicel) than below (in the stem fragment) prevents organs abscission [38]. All factors perturbing the natural balance of this hormone, e.g., artificial AZ activation by organ removal, direct IAA application, or treatment with TIBA (2,3,5-triiodobenzoic acid, polar auxin transport inhibitor), activate the AZ [6,7,39,40]. A similar effect is observed here, in our presented investigation, in which we provide novel facts about auxin involvement in stress-induced abscission processes. Once we applied drought conditions, the endogenous content of IAA in the floral lupine AZ increased (Figure 2G). This is a manifestation of disruption of the IAA natural balance, which is a reason for AZ activation. The hormone was localized in round structures presented in AZ cells (Figure 2B). We hypothesized that it could be endoplasmic reticulum. PIN5, PIN6, and PIN8 (PIN-FORMED) belonging to the family of transmembrane proteins that transport auxin across the plasma membrane are also located in this compartment and have been proposed to function in the regulation of cellular auxin homeostasis [41,42,43,44]. Presented here, data show that another prominent cell compartment in AZ cells, which accumulates auxin, is the nucleus (Figure 2C). It could support that the induction of the IAA signaling pathway is stimulated, as that auxin receptor TIR1 (TRANSPORT INHIBITOR RESPONSE 1) is active in its auxin-bound form within nuclei, inducing transcription of genes regulated by auxin [45]. As shown in the fruits of *Mangifera indica* and *Solanum lycopersicum*, IAA transport is negatively regulated by ET [46,47]. We have recently proven the stimulatory role of drought on the ET biosynthesis pathway locally in floral AZ cells [3]. Therefore, it cannot be excluded that the IAA accumulation, presented here, during the drought in the AZ (Figure 2B) is evoked by formed ET [3]. On the other hand, there are some reports suggesting the enhanced sensitivity of AZ cells in response to the disruption of natural IAA balance in different plant species [38,48], including lupine [6,7]. It is even more complicated given the case of *Populus*, in which IAA acts independently of ET in organ abscission [49].

Auxin has been shown to act in parallel and independently of ET on the hydrolysis of middle lamella [49,50]. The cell wall is exposed to high hydrostatic pressure induced by water deficit and, as a consequence, its structure is interrupted. The main factor determining the flexibility of cell walls is polysaccharides, including pectin, which, as the main component of the middle lamella, determines cell adhesion. The drought-induced changes in the pectin content (Figure 2) and the degree of their methylation (Figure 3) in the AZ of lupine flowers indicate activation of this structure. Importantly, the accumulation of pectin (Figure 2D) may suggest that they participate in the formation of new cell walls formed as a result of intensive divisions (Supplementary Figure S1E,F). On the other hand, the specific distribution pattern of methylated pectin (Figure 4) in response to drought indicates an intensive reorganization of the cell wall structure. High-methylated pectin, characteristic for new cell walls [51], can undergo de-methyl esterification catalyzed by PME, which was specifically localized in the AZ (Figure 5B,C). Reactions of the released carboxyl groups with Ca^2+^ ions affect the cell wall properties, its porosity, and ionic state [52]. Most plant PMEs remove methyl esters in a blocky way to form long chains of de-esterified pectin [53]. Analyzing these data, it can be concluded that the drought stress leads to a strong, AZ-specific accumulation of low-methylated pectin (Figure 4E–G), which is preceded by the upregulation of high-methylated pectin (Figure 4H–J) and PME abundance. Successive release of HG methyl residues provides the necessary products for the action of PG, which are integral parts of cell wall remodeling events of the organ separation [54]. Our findings revealed that drought causes the accumulation of PG in the cytoplasm of AZ cells (Figure 5E,F). This process is associated with the degradation of the middle lamella and reorganization of the cell wall structure (Supplementary Figure S1C,E,D,F). A similar pectin distribution pattern to those observed in this paper occurs in the AZ during the flower abortion of *E. pulcherrima* [55]. Interestingly, different results were obtained for *S. lycopersicum*, in which, both before and during flower separation, these two types of methylated pectin have not been observed [56]. Leaf abscission in *Azolla* is accompanied by a decrease in the level of low-methylated pectin in the AZ, while palm oil fruit abortion upregulates these pectin contents [23,55,57,58]. Furthermore, studies on *Cucumis melo* revealed that fruit separation accelerates the transcriptional activity of the *MPG1* and *MPG2* genes exclusively in AZ cells [20]. Summarizing this part, it can be concluded that although several reports describe pectin distribution following abscission processes, the results obtained for lupine are the first indicating AZ-restricted pectin reorganization in response to drought, which provides important new insight into the mechanisms of organ separation under abiotic stress conditions.

Xyloglucans, the main hemicelluloses found of a dicotyledon cell wall, are strongly accumulated during abscission in *S. lycopersicum* [56] and *E. pulcherrima* flowers [55]. It has been proposed that xyloglucans may be substrates for cell wall-degrading enzymes and/or act as protective substances that appear in response to AZ activation [55,56]. The possibility of HG binding to xyloglucans via RG-I, including arabinans and galactans, has also been postulated [59,60,61]. The cellulose and hemicellulose pectin have been shown to play a key role in modulating cell wall structure in response to drought stress. As in the case of yellow lupine (Figure 6), decreasing cellulose levels in response to water deficit were also observed in *Arabidopsis*, tobacco suspension cells, grape leaves, and wheat roots [62]. On the other hand, research on cotton shows that the genes related to the biosynthesis of cellulose are expressed more intensively under drought [54], suggesting a species-dependent response to these stress conditions. In addition, a larger increase in cellulose content was observed as a result of severe drought stress in *Picea glauca* [63]. The decreasing hemicellulose levels in the lupine AZ may confirm the loss of integrity of the cell wall, which reduces its ability to counteract the increasing pressure of cell turgor in response to drought.

From our data, it can be concluded that drought disrupts the natural balance of IAA across the AZ that evokes specific, AZ-restricted changes in cell wall composition, leading to the whole flower abortion. Future studies should focus on the advancement of knowledge on possible new factors that could maintain auxin movement and stop the demethylation of pectin under drought-induced abscission. It provides an excellent basis for understanding the mechanisms of plant tolerance to unpredictable environmental conditions that determine the yield of economically important species.

## 4. Material and Methods 

### 4.1. Plant Material, Growth Conditions

The experiments were carried out on the abscission zone (AZ) of yellow lupine (*Lupinus luteus* L.) flowers (Supplementary Figure S2). For the analyses, we used seeds of epigonal variety (Taper), provided by Poznań Plant Breeding (Tulce, Poland). Immediately before the sowing, seeds were treated with antifungicide (Sarfun T 65 DS, 250 mL for 100 kg of seeds), and inoculated with the bacterial vaccine (Nitragina, Biofood S.C., Wałcz, Poland). Seeds were sown to 11 L pots (8 seeds per pot, 20 cm spaces, ± 5 cm depth) to RV class soil material from Zelgno (Poland). The 200 plants were grown in phytotrons under controlled light conditions (16 h of light + 8 h dark, white cool fluorescent lamps with total power stream of 130 µmol m^−2^ s^–1^) and temperature (21–23 °C). The plants were grown for 5 weeks in optimal 70% soil water holding capacity (WHC) and watered an equal amount for every pot—the quantity was adapted to the developmental state of plants. After that, all plants were divided into two groups: (1) Control growing for 2 weeks in 70% WHC; (2) drought-treated lupines growing for 2 weeks in 25% WHC. These conditions have already been optimized and described in our previous work [3]. For WHC calculation, the method of [63] modified by Wilmowicz et al. [3] was used. All analyses were performed on the AZ fragments. They were excised by a razor blade approx. 1 mm above the AZ (distal part, near pedicel) and below the AZ (proximal part, near stem) (for details, see Supplementary Figure S1). Drought-treated AZ fragments were collected on the 48th day of development. At the same time, we harvested the control (inactive AZ) given the morphological and anatomical criteria specified by microscopic analysis [64]. Plant material, which was used for determination of the content of proline, pectin, and hemicelluloses was frozen in liquid nitrogen and stored at –80 °C until reuse. For microscopic analysis, AZ fragments were immediately fixed to avoid tissue degradation (see the next section). 

### 4.2. Material Fixation

Plant material for microscopy analyses was placed in a fixative solution composed of: 4% paraformaldehyde (*w/v*)/0.25% glutaraldehyde (*v/v*) in 1x PBS buffer (pH 7.2) supplemented with 3% EDAC (N-(3-dimethylaminopropyl)-N′-ethylcarbodiimide hydrochloride) (*w/v*), which ensures phytohormones localization [3,6,7,65]. Samples were left for 12 h at 4 °C; then, the fixation was washed three times for 10 min in 1x PBS at RT (room temperature). In the next step, tissues were dehydrated using ethanol in rising concentrations: 30% EtOH (ethylene) + DTT (ditiotreitol) for 1 h at RT, 50% EtOH + DTT for 1 h at RT, 70% EtOH + DTT overnight at 4 °C, 90% EtOH + DTT for 1 h at RT, and 100% EtOH + DTT for 1 h at RT. Tissues were then supersaturated with BMM resin (20 mL butyl methacrylate, 5 mL methyl methacrylate, 0.125 g benzoin ethyl ether, and 0.0385 g DDT) surfeited with gas nitrogen. Tissues were supersaturated with changing concentrations of BMM (25%, 50%, 75%) in 99.8% EtOH (each step for 24 h at 4 °C). In the last stage, tissues were placed in 100% BMM solution at 4 °C for 24 h. The material was embedded in BEEM capsules. The polymerization process proceeded at −20 °C under UV light for 3 days. Tissues were cut to semi-thin sections (1.5 μm) using a Leica Ultramicrotome (Reichert-Jung, Wetzlar, Germany) and the material was then placed on glass slides covered with BioBond (BBInternational, Cardiff, UK).

### 4.3. Histochemical Analysis of AZ Structure

For general observations of AZ cells, sections were stained with 0.05% Toluidine blue solution for 10 min. Un-esterified pectin was detected with 0.02% (*w/v*) Ruthenium red ([(NH3)5Ru-O-Ru(NH3)4-O-Ru(NH3)5]Cl6) solution [66]. This dye was served for 30 min and the specimens were then washed with distilled water and observed using a microscope (LM Zeiss Axioplan, Oberkochen, Germany) with a ProGres C3 digital camera.

### 4.4. Immunocytochemical Assay

The BMM resin was removed from sections on slides by washing in 100% acetone (2 times for 20 min), distilled water (twice for 3 min), and 1x PBS buffer (5 min). After rinsing, AZ fragments were incubated with primary antibodies JIM5 (Cat. No. JIM5) and JIM7 (Cat. No. JIM7) (Plant Probes, Leeds, UK) dissolved in 1:20 in 1x PBS buffer with 1% Bovine Serum Albumin (BSA). High moisture and optimal temperature for incubation (4 °C, 24 h) were ensured. After this time, sections were washed with 1x PBS (3 times for 10 min). Next, they were incubated in a wet chamber with secondary antibodies (Anti-Rat conjugated with FITC, Ab6840, Abcam, Cambridge, UK) at 37 °C for 2 h. IAA localization was performed following the same conditions described in our papers [6,7]. After the last washing in 1x PBS buffer for 5 min, all obtained sections were incubated with DAPI for nuclei visualization. Then, the dye was washed by 1 x PBS for 10 min. Finally, an antifadant medium (MOWIOL with 2.5% DABCO) was applied directly to the sections [67]. Negative control was performed by omission of incubation with primary antibodies (Supplementary Figure S3). The results were documented using a fluorescent microscope (DM6000B, Leica, Wetzlar, Germany).

### 4.5. IAA GC-MS Analysis

For the purpose of measuring the endogenous level of IAA, gas chromatography-mass spectrometry (GC-MS) was used. The AZ fragments (0.5 g) were powdered and, subsequently, IAA content was analyzed according to protocols described by Wilmowicz et al. [5]. GC-MS-SIM was performed by monitoring m/z 130 for IAA-methyl ester and m/z 132 for deuterium-labeled IAA-methyl ester (d_2_-IAA).

### 4.6. Extraction and Determination of Pectin and Hemicellulose

The pectin was analyzed following Liu et al. [68]. The AZ fragments (~0.1 g) were weighed, rinsed with 0.5 mmol L^–1^ CaCl_2_ solution, and washed twice with deionized water. Next, tissues were placed in a mortar, 0.5 mL pre-cooled 75% EtOH was added, and the material was homogenized. Suspension of homogenate was transferred into 2.0 mL Eppendorf tubes and centrifuged (10,000× *g* for 10 min). The supernatant was discarded, while the precipitate was washed with 1 mL of methanol:chloroform mixture (1:1, *v/v*). Then, samples were centrifuged (10,000× *g* for 10 min), the supernatant was discarded, 1 mL of acetone was added, and the precipitation was mixed and subsequently freeze-dried. In the next step, 2 mL of ammonium-oxalate buffer was added to the extracted cell wall. The mixture was incubated in a water bath (97 °C for 1 h) and centrifuged (10,000× *g* for 10 min). The above procedure, starting from the ammonium-oxalate buffer step, was repeated twice, and both supernatants were combined and the pectin content was determined spectrophotometrically (280 nm) (Shimadzu Europe-UV mini-1240, Kyoto, Japan).

For hemicellulose detection, 2 mL of 4% KOH was added to the precipitate for 12 h of extraction. Next, the extract was centrifuged (10,000× *g* for 10 min) and the supernatant was collected. This procedure was repeated twice. The supernatants were combined as a firth fraction of hemicellulose. The above procedure was repeated and obtained extracts were regarded as the second fraction of hemicellulose. Both fractions were combined, and the absorbance of the supernatant was measured at 280 nm (see above) using the same equipment as for pectin.

### 4.7. Statistical Analysis

Obtained data are the results of three biological samples with two technical replicates (each biological sample was analyzed two times, *n* = 3) and presented as mean ± standard error (SE). The statistical analysis of the data from the determination of proline and IAA concentration, as well as pectin and hemicellulose absorbance, was performed in Microsoft Excel using the Student’s *t*-test. The SigmaPlot 2001 v.7.0 was used to generate the graphs.

## Supplementary Materials

The following are available online at https://www.mdpi.com/1422-0067/21/18/6848/s1.

## Abbreviations

ABA	abscisic acid
AZ	abscission zone
DIST	flower pedicel fragments above AZ
ET	ethylene
H_2_O_2_	hydrogen peroxide
HG	homogalacturonans
IAA	Indole-3-acetic acid
PG	polygalacturonase
PIN	PIN-FORMED
PME	pectin methylesterase
PROX	stem fragments below the AZ
RG	rhamnogalacturonans
ROS	reactive oxygen species
TIBA	2,3,5-triiodobenzoic acid
TIR1	TRANSPORT INHIBITOR RESPONSE 1
VB	vascular bundles
WHC	water holding capacity

## References

[1] McKeown A.W., Warland J., McDonald M.R. (2006). Long-term climate and weather patterns in relation to crop yield: A minireview. Botany.

[2] Samaniego L., Thober S., Kumar R., Wanders N., Rakovec O., Pan M., Marx A. (2018). Anthropogenic warming exacerbates European soil moisture droughts. Nat. Clim. Chang..

[3] Wilmowicz E., Kućko A., Burchardt S., Przywieczerski T. (2019). Molecular and hormonal aspects of drought-triggered flower shedding in yellow lupine. Int. J. Mol. Sci..

[4] Wilmowicz E., Kućko A., Ostrowski M., Panek K. (2018). *INFLORESCENCE DEFICIENT IN ABSCISSION-like* is an abscission-associated and phytohormone-regulated gene in flower separation of *Lupinus luteus*. Plant Growth Regul..

[5] Wilmowicz E., Frankowski K., Kućko A., Świdziński M., de Dios Alché J., Nowakowska A., Kopcewicz J. (2016). The influence of abscisic acid on the ethylene biosynthesis pathway in the functioning of the flower abscission zone in *Lupinus luteus*. J. Plant Physiol..

[6] Kućko A., Wilmowicz E., Ostrowski M. (2019). Spatio-temporal IAA gradient is determined by interactions with ET and governs flower abscission. J. Plant Physiol..

[7] Kućko A., Wilmowicz E., Pokora W., de Dios Alché J. (2020). Disruption of the auxin gradient in the abscission zone area evokes asymmetrical changes leading to flower separation in yellow lupine. Int. J. Mol. Sci..

[8] Tucker M.L., Sexton R., Del Campillo E., Lewis L.N. (1988). Bean abscission cellulase characterization of a cDNA clone and regulation of gene expression by ethylene and auxin. Plant Physiol..

[9] Ferrarese L., Trainotti L., Moretto P., de Laureto P.P., Rascio N., Casadoro G. (1995). Differential ethylene-inducible expression of cellulase in pepper plants. Plant Mol. Biol..

[10] Estornell L.H., Agusti J., Merelo P., Talon M., Tadeo F.R. (2013). Elucidating mechanisms underlying organ abscission. Plant Sci..

[11] Roberts J.A., Gonzalez-Carranza Z.H. (2009). Pectinase functions in abscission. Stewart Postharvest Rev..

[12] Ranjbar A., Ahmadi N. (2016). Effects of External Ethylene on Laccase and Antioxidant Enzymes Activity, and Physio-Biochemical. J. Hortic..

[13] Merelo P., Agustí J., Arbona V., Costa M.L., Estornell L.H., Gómez-Cadenas A., Coimbra S., Gomez M.D., Perez-Amador M.A., Domingo C. (2017). Cell wall remodeling in abscission zone cells during ethylene-promoted fruit abscission in citrus. Front. Plant Sci..

[14] Harholt J., Suttangkakul A., Scheller H.V. (2010). Biosynthesis of pectin. Plant Physiol..

[15] Atmodjo M.A., Hao Z., Mohnen D. (2013). Evolving views of pectin biosynthesis. Annu. Rev. Plant Biol..

[16] Daher F.B., Braybrook S.A. (2015). How to let go: Pectin and plant cell adhesion. Front. Plant Sci..

[17] Sénéchal F., Wattier C., Rustérucci C., Pelloux J. (2014). Homogalacturonan-modifying enzymes: Structure, expression, and roles in plants. J. Exp. Bot..

[18] Kalaitzis P., Koehler S.M., Tucker M.L. (1995). Cloning of a tomato polygalacturonase expressed in abscission. Plant Mol. Biol..

[19] Wolf S., Mouille G., Pelloux J. (2009). Homogalacturonan methyl-esterification and plant development. Mol. Plant.

[20] Hadfield K.A., Bennett A.B. (1998). Polygalacturonases: Many genes in search of a function. Plant Physiol..

[21] Ogawa M., Kay P., Wilson S., Swain S.M. (2009). ARABIDOPSIS DEHISCENCE ZONE POLYGALACTURONASE1 (ADPG1), ADPG2, and QUARTET2 are polygalacturonases required for cell separation during reproductive development in *Arabidopsis*. Plant Cell.

[22] Swain S., Kay P., Ogawa M. (2011). Preventing unwanted breakups: Using polygalacturonases to regulate cell separation. Plant Signal. Behav..

[23] Roongsattham P., Morcillo F., Fooyontphanich K., Jantasuriyarat C., Tragoonrung S., Amblard P., Collin M., Mouille G., Verdeil J.-L., Tranbarger T.J. (2016). Cellular and pectin dynamics during abscission zone development and ripe fruit abscission of the monocot oil palm. Front. Plant Sci..

[24] Hayat S., Hayat Q., Alyemeni M.N., Wani A.S., Pichtel J., Ahmad A. (2012). Role of proline under changing environments: A review. Plant Signal. Behav..

[25] Majda M., Robert S. (2018). The role of auxin in cell wall expansion. Int. J. Mol. Sci..

[26] Willats W.G., Limberg G., Buchholt H.C., Alebeek G.J.V., Benen J., Christensen T.M., Visser J., Voragen A., Mikkelsen J.D., Knox J.P. (2000). Analysis of pectic epitopes recognised by hybridoma and phage display monoclonal antibodies using defined oligosaccharides, polysaccharides, and enzymatic degradation. Carbohydr. Res..

[27] Wakabayashi S., Shigekawa M., Pouyssegur J. (1997). Molecular physiology of vertebrate Na^+^/H^+^ exchangers. Physiol. Rev..

[28] Somerville C., Bauer S., Brininstool G., Facette M., Hamann T., Milne J., Osborne E., Paredez A., Persson S., Raab T. (2004). Toward a systems approach to understanding plant cell walls. Science.

[29] Szabados L., Savoure A. (2010). Proline: A multifunctional amino acid. Trends Plant Sci..

[30] Sharma P., Jha A.B., Dubey R.S., Pessarakli M. (2012). Reactive oxygen species, oxidative damage, and antioxidative defense mechanism in plants under stressful conditions. J. Bot..

[31] Öztürk L., Demir Y. (2002). In Vivo and in vitro protective role of proline. Plant Growth Regul..

[32] Orlikowska T., Kucharska D., Horbowicz M. (2009). The reaction of raspberry and blackberry cultivars to drought stress simulated in vitro by polyethylene glycol (PEG) 6000. Acta Hortic..

[33] Jouve L., Hoffmann L., Hausman J.F. (2004). Polyamine, carbohydrate, and proline content changes during salt stress exposure of aspen (*Populus tremula* L.): Involvement of oxidation and osmoregulation metabolism. Plant Biol..

[34] Verbruggen N., Villarroel R., Van Montagu M. (1993). Osmoregulation of a pyrroline-5-carboxylate reductase gene in *Arabidopsis thaliana*. Plant Physiol..

[35] Dawood M.G., Taie H.A.A., Nassar R.M.A., Abdelhamid M.T., Schmidhalter U. (2014). The changes induced in the physiological, biochemical and anatomical characteristics of *Vicia faba* by the exogenous application of proline under seawater stress. S. Afr. J. Bot..

[36] Jie Z., Yuncong Y., Streeter J.G., Ferree D.C. (2010). Influence of soil drought stress on photosynthesis, carbohydrates and the nitrogen and phophorus absorb in different section of leaves and stem of Fugi/M. 9EML, a young apple seedling. Afr. J. Biotechnol..

[37] Chaitanya K.V., Rasineni G.K., Reddy A.R. (2009). Biochemical responses to drought stress in mulberry (*Morus alba* L.): Evaluation of proline, glycine betaine and abscisic acid accumulation in five cultivars. Acta Physiol. Plant..

[38] Louie D.S.J., Addicott F.T. (1970). Applied auxin gradients and abscission in explants. Plant Physiol..

[39] Sexton R., Roberts J.A. (1982). Cell biology of abscission. Annu. Rev. Plant Physiol..

[40] Abeles F.B., Rubinstein B. (1964). Regulation of ethylene evolution and leaf abscission by auxin. Plant Physiol..

[41] Mravec J., Skůpa P., Bailly A., Hoyerová K., Křeček P., Bielach A., Petrášek J., Zhang J., Gaykova V., Stierhof Y.D. (2009). Subcellular homeostasis of phytohormone auxin is mediated by the ER-localized PIN5 transporter. Nature.

[42] Bosco C.D., Dovzhenko A., Liu X., Woerner N., Rensch T., Eismann M., Eimer S., Hegermann J., Paponov I.A., Ruperti B. (2012). The endoplasmic reticulum localized PIN8 is a pollen-specific auxin carrier involved in intracellular auxin homeostasis. Plant J..

[43] Ding Z., Wang B., Moreno I., Dupláková N., Simon S., Carraro N., Reemmer J., Pěnčík A., Chen X., Tejos R. (2012). ER-localized auxin transporter PIN8 regulates auxin homeostasis and male gametophyte development in Arabidopsis. Nat. Commun..

[44] Simon S., Skůpa P., Dobrev P.I., Petrášek J., Zažímalová E., Friml J. (2014). Analyzing the in vivo status of exogenously applied auxins: A HPLC-based method to characterize the intracellularly localized auxin transporters. Methods Mol. Biol..

[45] Dharmasiri N., Dharmasiri S., Weijers D., Lechner E., Yamada M., Hobbie L., Ehrismann J.S., Jürgens G., Estelle M. (2005). Plant development is regulated by a family of auxin receptor F box proteins. Dev. Cell.

[46] Hagemann M.H., Winterhagen P., Hegele M., Wünsche J.N. (2015). Ethephon induced abscission in mango: Physiological fruitlet responses. Front. Plant Sci..

[47] Shi J., Dong J., Xue J., Wang H., Yang Z., Jiao Y., Xu L., Huang H. (2017). Model for the role of auxin polar transport in patterning of the leaf adaxial–abaxial axis. Plant J..

[48] Addicott F.T., Lynch R.S., Carns H.R. (1955). Auxin gradient theory of abscission regulation. Science.

[49] Jin X., Zimmermann J., Polle A., Fischer U. (2015). Auxin is a long-range signal that acts independently of ethylene signaling on leaf abscission in *Populus*. Front. Plant Sci..

[50] Bleecker A.B., Patterson S.E. (1997). Last exit: Senescence, abscission, and meristem arrest in Arabidopsis. Plant Cell.

[51] Kim J., Sundaresan S., Philosoph-Hadas S., Yang R., Meir S., Tucker M.L. (2015). Examination of the abscission-associated transcriptomes for soybean, tomato, and *Arabidopsis* highlights the conserved biosynthesis of an extensible extracellular matrix and boundary layer. Front. Plant Sci..

[52] Leroux O., Knox J.P., Leroux F., Vrijdaghs A., Bellefroid E., Borgonie G., Viane R.L. (2007). Intercellular pectic protuberances in *Asplenium*: New data on their composition and origin. Ann. Bot..

[53] Limberg G., Körner R., Buchholt H.C., Christensen T.M., Roepstorff P., Mikkelsen J.D. (2000). Analysis of different de-esterification mechanisms for pectin by enzymatic fingerprinting using endopectin lyase and endopolygalacturonase II from *A. niger*. Carbohydr. Res..

[54] Roongsattham P., Morcillo F., Jantasuriyarat C., Pizot M., Moussu S., Jayaweera D., Collin M., Conzales-Carranza Z.H., Amblard P., Tregear J.M. (2012). Temporal and spatial expression of polygalacturonase gene family members reveals divergent regulation during fleshy fruit ripening and abscission in the monocot species oil palm. BMC Plant Biol..

[55] Lee Y., Derbyshire P., Knox J.P., Hvoslef-Eide A.K. (2008). Sequential cell wall transformations in response to the induction of a pedicel abscission event in *Euphorbia pulcherrima* (poinsettia). Plant J..

[56] Iwai H., Terao A., Satoh S. (2013). Changes in distribution of cell wall polysaccharides in floral and fruit abscission zones during fruit development in tomato (*Solanum lycopersicum*). J. Plant. Res..

[57] Uheda E., Nakamura S. (2000). Abscission of *Azolla* branches induced by ethylene and sodium azide. Plant Cell Physiol..

[58] Bowling A.J., Vaughn K.C. (2011). Leaf abscission in *Impatiens* (*Balsaminaceae*) is due to loss of highly de-esterified homogalacturonans in the middle lamellae. Am. J. Bot..

[59] Ridley B.L., O’Neill M.A., Mohnen D. (2001). Pectins: Structure, biosynthesis, and oligogalacturonide-related signaling. Phytochemistry.

[60] Popper Z.A., Fry S.C. (2008). Xyloglucan—Pectin linkages are formed intra-protoplasmically, contribute to wall-assembly, and remain stable in the cell wall. Planta.

[61] Wefers D., Tyl C.E., Bunzel M. (2014). Novel arabinan and galactan oligosaccharides from dicotyledonous plants. Front. Chem..

[62] Feng W., Lindner H., Robbins N.E., Dinneny J.R. (2016). Growing out of stress: The role of cell-and organ-scale growth control in plant water-stress responses. Plant Cell.

[63] Chauhan B.S., Johnson D.E. (2011). Growth response of direct-seeded rice to oxadiazon and bispyribac-sodium in aerobic and saturated soils. Weed Sci..

[64] Frankowski K., Wilmowicz E., Kućko A., Zienkiewicz A., Zienkiewicz K., Kopcewicz J. (2015). Profiling the *BLADE-ON-PETIOLE* gene expression in the abscission zone of generative organs in *Lupinus luteus*. Acta Physiol. Plant..

[65] Ondzighi-Assoume C.A., Chakraborty S., Harris J.M. (2016). Environmental nitrate stimulates abscisic acid accumulation in *Arabidopsis* root tips by releasing it from inactive stores. Plant Cell.

[66] Sabba R.P., Lulai E.C. (2002). Histological analysis of the maturation of native and wound periderm in potato (*Solanum tuberosum* L.) tuber. Ann. Bot..

[67] Płachno B., Lubomír A., Świątek P., Kapusta M., Miranda V.F.O. (2020). Life in the current: Anatomy and morphology of *Utricularia neottioides*. Int. J. Mol. Sci..

[68] Liu Z., Pi F., Guo X., Guo X., Yu S. (2019). Characterization of the structural and emulsifying properties of sugar beet pectins obtained by sequential extraction. Food Hydrocol..

