# Supplementary material for: Drought Disrupts Auxin Localization in Abscission Zone and Modifies Cell Wall Structure Leading to Flower Separation in Yellow Lupine
Source: Int J Mol Sci. 2020 Sep 18;21(18):6848. doi: 10.3390/ijms21186848 (PMC7555076; doi:10.3390/ijms21186848)
Supplement: Supplementary file 1 [file ijms-21-06848-s001.pdf]

Acronyms used in the text

**ABBREVIATIONS** in alphabetical order	**DEFINITION**
**ABA**	abscisic acid
**AZ**	abscission zone
**DIST**	flower pedicel fragments above the az
**ET**	ethylene
**HG**	homogalacturonans
**IAA**	indole-3-acetic acid
**PG**	polygalacturonase
**PME**	pectin methylesterase
**PROX**	stem fragments below the AZ
**VB**	vascular bundles
**WHC**	water holding capacity

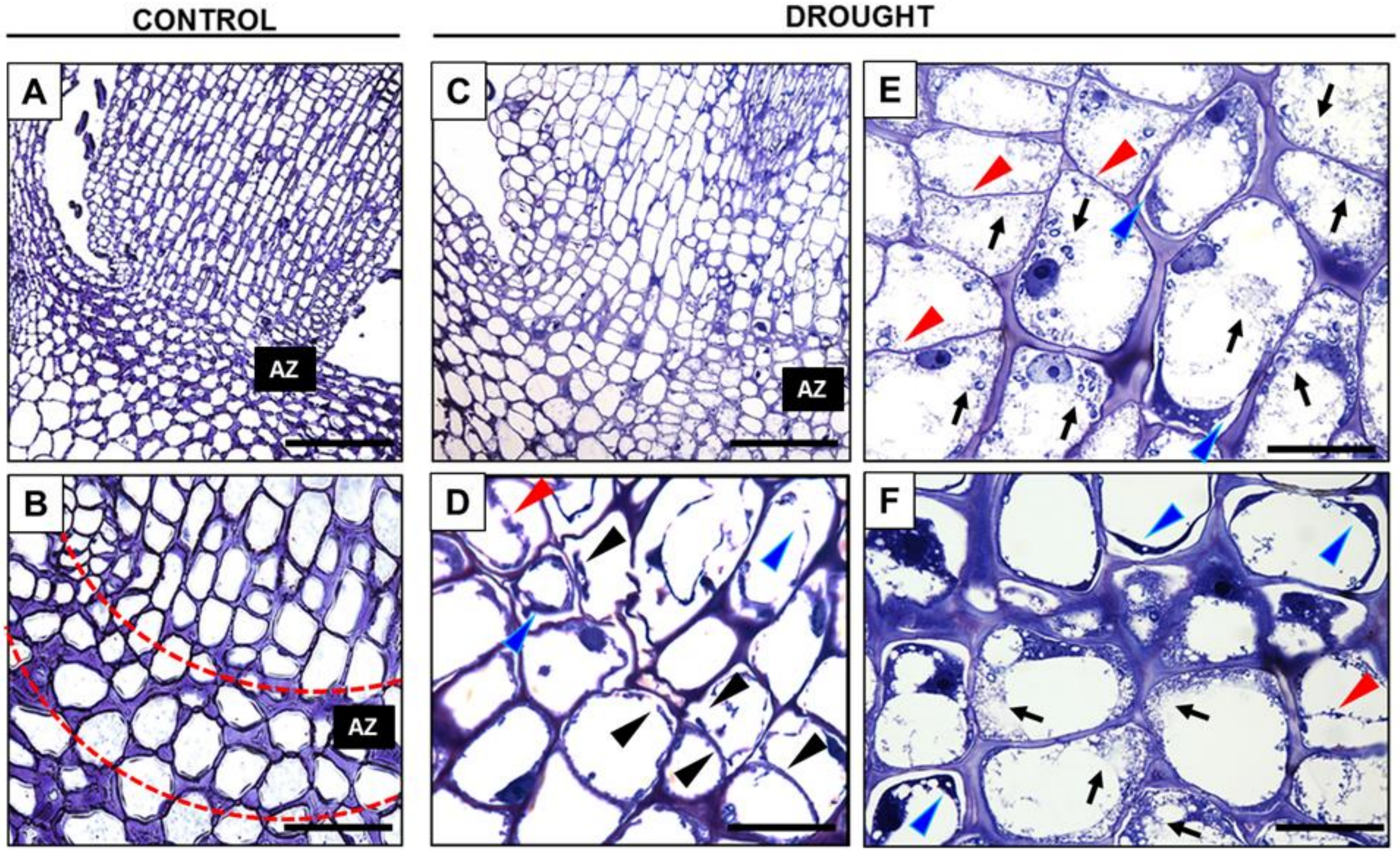

Supplementary Figure S1. Drought stress evokes specific cellular changes in the floral AZ of *Lupinus luteus*. For histological observations, sections of abscission zone (AZ) were collected on the 48th day of cultivation from control plants growing in the soil of optimal moisture (75% WHC) (**A,B**) and drought-treated ones (25% WHC) (**C,E,F**). Fixed material was stained with toluidine blue. Picture B presents a magnified region of AZ from A, while and D, E, F corresponds to different, magnified areas of AZ from the C. AZ area is marked by red dotted lines (**B**). Blue arrowheads indicate shrunken protoplast (**D,E,F**), red arrowheads mark the place of newly formed cell walls after divisions (**D,E,F**), black arrows show the presence of aggregates and small vesicles in the cytoplasm (**E,F**), whereas the black arrowheads indicate a place of the middle lamella hydrolysis (**D**). Bars – 200 μm (A,C), 50 μm (B), 20 μm (D,E,F).

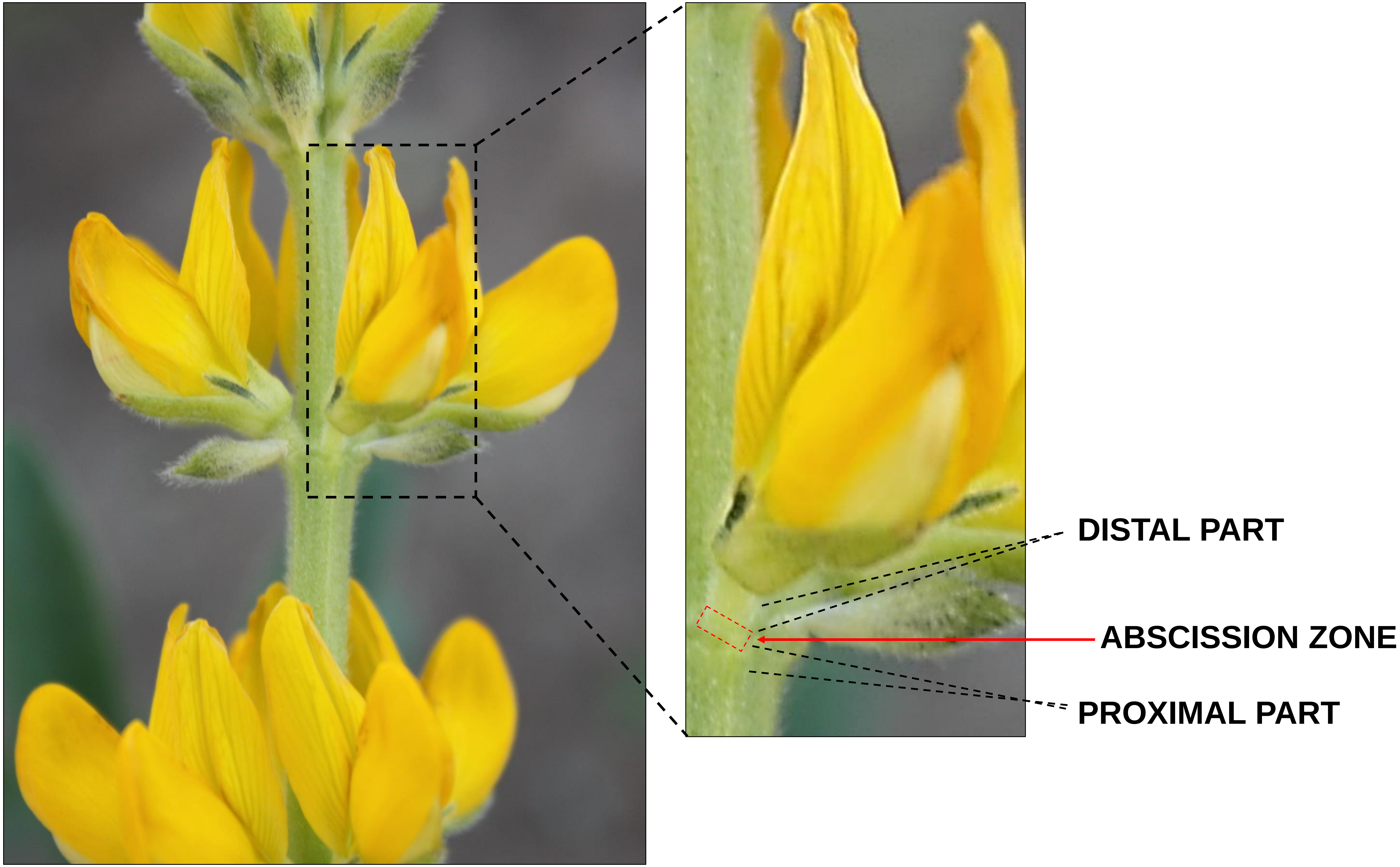

Supplementary Figure S2. Flower of *Lupinus luteus* L. Tissue fragments (indicated by red boxes) containing abscission zone (AZ) dissected 1 mm above (distal part) and below AZ (proximal part) were collected for analyses.

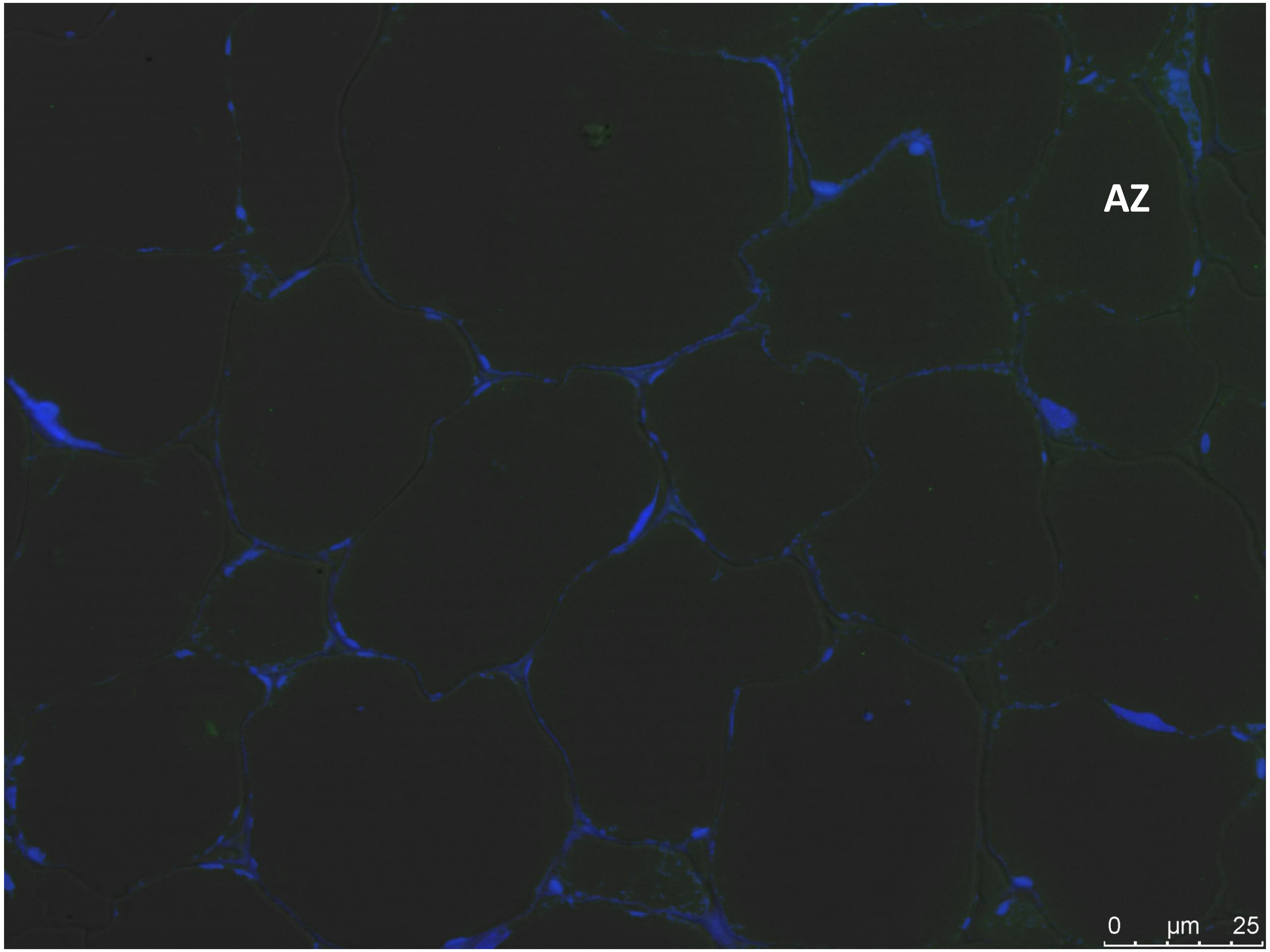

Supplementary Figure 3. The results of a control reaction performed with omitting primary antibodies anti-IAA, anti-JIM5, anti-JIM7, anti-PME, and anti-PG. Control reaction produced negative results compared with those of standard reactions. Nuclei were stained with DAPI. Abbreviation: AZ – abscission zone.
